# Data-driven segmentation of audiometric phenotypes across a large clinical cohort

**DOI:** 10.1038/s41598-020-63515-5

**Published:** 2020-04-21

**Authors:** Aravindakshan Parthasarathy, Sandra Romero Pinto, Rebecca M. Lewis, William Goedicke, Daniel B. Polley

**Affiliations:** 1 Eaton-Peabody Laboratories, Department of Otolaryngology – Head and Neck Surgery, Massachusetts Eye and Ear, Boston, MA 02114 USA; 2 Department of Otolaryngology – Head and Neck Surgery, Harvard Medical School, Boston, MA 02114 USA; 3 Present Address: National Military Audiology and Speech Pathology Center, Walter Reed National Military Medical Center, Bethesda, MD 20889 USA

**Keywords:** Cochlea, Diagnostic markers

## Abstract

Pure tone audiograms are used to assess the degree and underlying source of hearing loss. Audiograms are typically categorized into a few canonical types, each thought to reflect distinct pathologies of the ear. Here, we analyzed 116,400 patient records from our clinic collected over a 24-year period and found that standard categorization left 46% of patient records unclassified. To better account for the full spectrum of hearing loss profiles, we used a Gaussian Mixture Model (GMM) to segment audiograms without any assumptions about frequency relationships, interaural symmetry or etiology. The GMM converged on ten types, featuring varying degrees of high-frequency hearing loss, flat loss, mixed loss, and notched profiles, with predictable relationships to patient age and sex. A separate GMM clustering of 15,380 audiograms from the National Health and Nutrition Examination Survey (NHANES) identified six similar types, that only lacked the more extreme hearing loss configurations observed in our patient cohort. Whereas traditional approaches distill hearing loss configurations down to a few canonical types by disregarding much of the underlying variability, an objective probabilistic model that accounted for all of the data identified an organized, but more heterogenous set of audiogram types that was consistent across two large clinical databases.

## Introduction

The pure tone audiogram is the current gold standard clinical hearing assessment. Clinical management of hearing loss, from diagnosis to intervention, largely depends upon quantifying pure tone thresholds at octave intervals between 250 and 8000 Hz^1^. Multiple factors including age, genetic background and noise exposure history can influence the level and configuration of pure-tone thresholds within the standard audiometric frequency range^2–5^.

A long-standing goal has been to infer the underlying cause of hearing loss using the pure-tone audiogram, medical diagnoses and knowledge from post-mortem human temporal bone and animal studies^6–10^. These approaches have identified four essential types - (*i*) a normal audiogram, where thresholds across test frequencies are equal or better than 20 dB HL, (*ii*) a flat hearing loss, where audiogram thresholds are elevated outside of the normal range but are approximately flat across test frequencies, presumably due to possible metabolic losses in the stria vascularis; (*iii*) a sloping, high frequency hearing loss (HFHL) due to presumed sensorineural damage; and (*iv*) a mixed phenotype reflecting modest threshold shift at low frequencies and a steeply sloping loss at high frequencies, which has been interpreted as a mixture of metabolic and sensorineural contributors^7,11–15^.

These categories are useful as a first approximation, but studies with larger data samples either note the presence of mixed phenotypes or phenotypes with indeterminate causes^6^. Studies using algorithmic clustering methods note the presence of a gradual continuum of shapes rather than distinct categories^16^. Additionally, a recent study that reimaged historical temporal bone data has called into question the role of the stria vascularis in producing flat audiograms, and suggests that all audiogram shapes may in fact be driven primarily by outer hair cell loss^17^.

Optimal classification of audiometric phenotypes will inform more nuanced investigations of the causes of underlying etiology, ultimately facilitating individualized therapeutic options for patients by identifying markers for threshold shifts that go beyond cochlear pathology. In this study, instead of classification methods reflecting presumed – but ultimately unverifiable – inner ear pathologies we sought to apply a purely data-driven classification approach. By design, we investigated classification strategies that segmented audiogram profiles into types based on the naturally occurring patterns of inherent variability without any knowledge of how thresholds for a particular frequency relate to other frequencies, the other ear, or patient demographics, and describe their prevalence from large samples of clinical and normative subjects.

## Methods

### Study population

#### Massachusetts Eye and Ear audiology database

**Figure 1:**
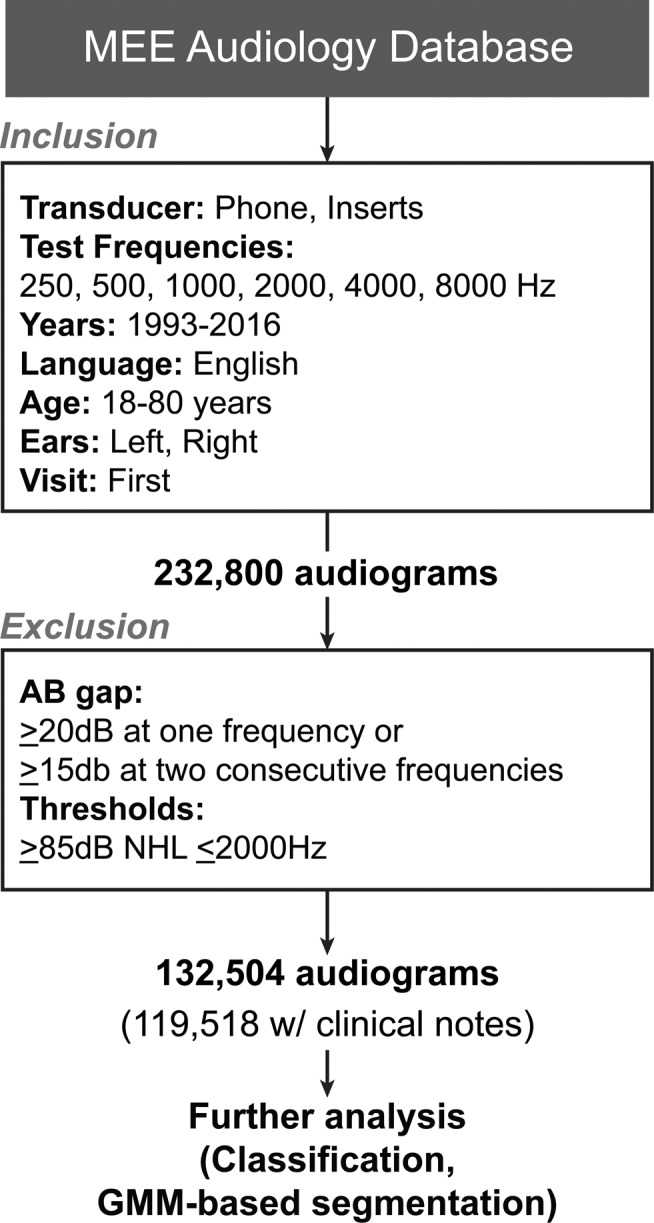
Inclusion and exclusion criteria for patients from the audiology database of the Massachusetts Eye and Ear, Boston, MA, USA.

We analyzed first-visit patient records from the MEE audiology database over a 24-year period from 1993 to 2016. The inclusion criteria, including age range, primary language, test frequencies and transducers used to test threshold sensitivity are given in Fig. 1. This resulted in 232,800 audiograms from 116,400 patients with data for the six test frequencies (250 Hz to 8000 Hz at octave spacing) from both ears to be included in the study. To eliminate patients with conductive components in their hearing loss, the MEE dataset was further curated to remove all audiograms where the air-bone gap was ≥20 dB at any one frequency or ≥15 dB at two consecutive frequencies. Audiograms with thresholds ≥85 dB normalized hearing level (nHL) at frequencies ≤2000 Hz were also removed to maintain a conservative inclusion criterion, as the difference in limits of the air and bone conducting transducers limit our ability to determine the presence of conductive components in that threshold range. After this exclusionary step, we were left with 132,504 audiograms in the MEE dataset for analysis (Fig. 1). From this cohort, clinical notes describing the primary reason for visit were only available beginning in the year 2000. This winnowed down our dataset to 119,518 audiograms between 2000 and 2016 for the portions of the manuscript dealing with clinical notes. The study was approved by the human subjects Institutional Review Board at the Massachusetts Eye and Ear. Requirements of informed consent were waived for this retrospective chart review by the Institutional Review Board at the Massachusetts Eye and Ear. Data analysis was performed on de-identified data, in accordance with the relevant guidelines and regulations.

#### National health and nutrition examination survey (NHANES) database

The National Health and Nutrition Examination Survey (NHANES) is a nationally representative cross-sectional survey of civilian non-institutionalized adults and children in the United States, conducted by the National Center for Health Statistics. As part of NHANES, high quality audiometric thresholds were collected at seven test frequencies, 500 Hz, 1000 Hz, 2000 Hz, 3000 Hz, 4000 Hz, 6000 Hz and 8000 Hz, by trained health technicians in mobile sound isolated examination rooms, using an standard audiological testing equipment (audiometer: AD 226, Interacoustics AS; headphones: TDH 39, Telephonics; earphones: EarTone 3A, Etymotic Research). Audiological data from seven NHANES datasets between 1999 and 2012 were downloaded from the National Centre for Health statistics website (www.cdc.gov/nchs/nhanes/) and merged into a single dataset. Audiometric protocols and details of data collection are available on the NCHS website. Audiograms from left and right ears, of adults between 18–80 years were compiled using custom MATLAB scripts. This provided an initial set of 15,380 audiograms (7688 from the left ear) from the NHANES database.

### Clustering using gaussian mixture models

#### Data preprocessing

We used the same test frequencies available in the NHANES dataset for the MEE dataset (500, 1000, 2000, 3000, 4000, 6000 and 8000 Hz). Since measuring inter-octaves is not standard clinical practice, we performed a linear interpolation of all the audiograms at 3000 Hz and 6000 Hz test frequencies in the MEE dataset to account for the missing data points. We also removed audiograms with thresholds ≥85 dB NHL at frequencies at and below 2000 Hz in the NHANES dataset to match the exclusion criteria of the MEE dataset. After this preprocessing, we were left with 15,340 audiograms from left and right ears in the NHANES dataset and 132,504 audiograms from left and right ears in the MEE dataset being used for clustering using the Gaussian mixture models.

#### Gaussian mixture model

Clustering was performed with Gaussian mixture models (GMM) using the Statistics Toolbox in MATLAB 2017b. This is a probabilistic model that assumes the data are generated from a mixture of a finite number of multivariate Gaussian distributions. In comparison to simpler analog techniques such as k-means, GMM doesn’t assume an equal variance of each cluster and thus, incorporates information about the covariance structure of the data as well as the means of the latent Gaussians. Each cluster represents a category of samples (i.e. audiometric phenotype) and is defined as a multivariate Gaussian distribution composed of multidimensional vector measurements (i.e. individual audiograms). The sample dimensions were the audiometric thresholds at each test frequency. Each audiogram was then given a probability of belonging to each of the clusters (mixture coefficients), and assigned to the cluster with the maximum probability. The parameters are optimized by the mean and variances of each cluster, and the mixture coefficients, and therefore scale with the number of components and samples.

The GMM was initialized by selecting a range of 2–15 clusters, and computing the initial parameters using k-means clustering. We then used the expectation-maximization (EM) algorithm for iteratively fitting the optimal model, starting from the initial parameter values and performing an E-step and an M-step until convergence is detected. Given the initial Gaussian distributions, the probability that each data point belongs to a particular cluster is computed in the E-step. Based on these probabilities, a new set of parameters for the Gaussian distributions is computed such that the probabilities of data points within the clusters are maximized, in the M-Step. These steps are iteratively repeated until reaching convergence. We assumed a full, unshared covariance matrix, where each component has its own covariance matrix allowing for correlated predictors. The convergence criterion was set to having a change of 1e-6 in the log likelihood of the model fit between iterations. To avoid ill-conditioned (singular) covariance matrices due to correlations between dimensions, we used a regularization value of 0.01, which is added to the diagonal elements of the covariance matrices and ensures all matrices are positive-definite.

The criterion to choose the number of clusters was given by the Bayesian Information Criterion (BIC), for the model fits with each number of clusters. This information criterion considers the negative log-likelihood of the model and penalizes it with model complexity (number of parameters to fit). Minimization of the BIC value indicates a good model fit. We chose this as opposed to the Akaike information criterion (AIC), as the latter tends to choose more complex models that might overfit, whereas BIC is more conservative. However, we evaluated both criteria and observed similar saturation points in their values as a function of the number of clusters. These criteria are given by:

$$\begin{array}{c}AIC=2M-2LogL\\ BIC=Mlog(N)-2LogL\end{array}$$

where M is the number of parameters, N the number of samples and logL the log likelihood of the model fit.

## Results

### Patient demographics

**Figure 2:**
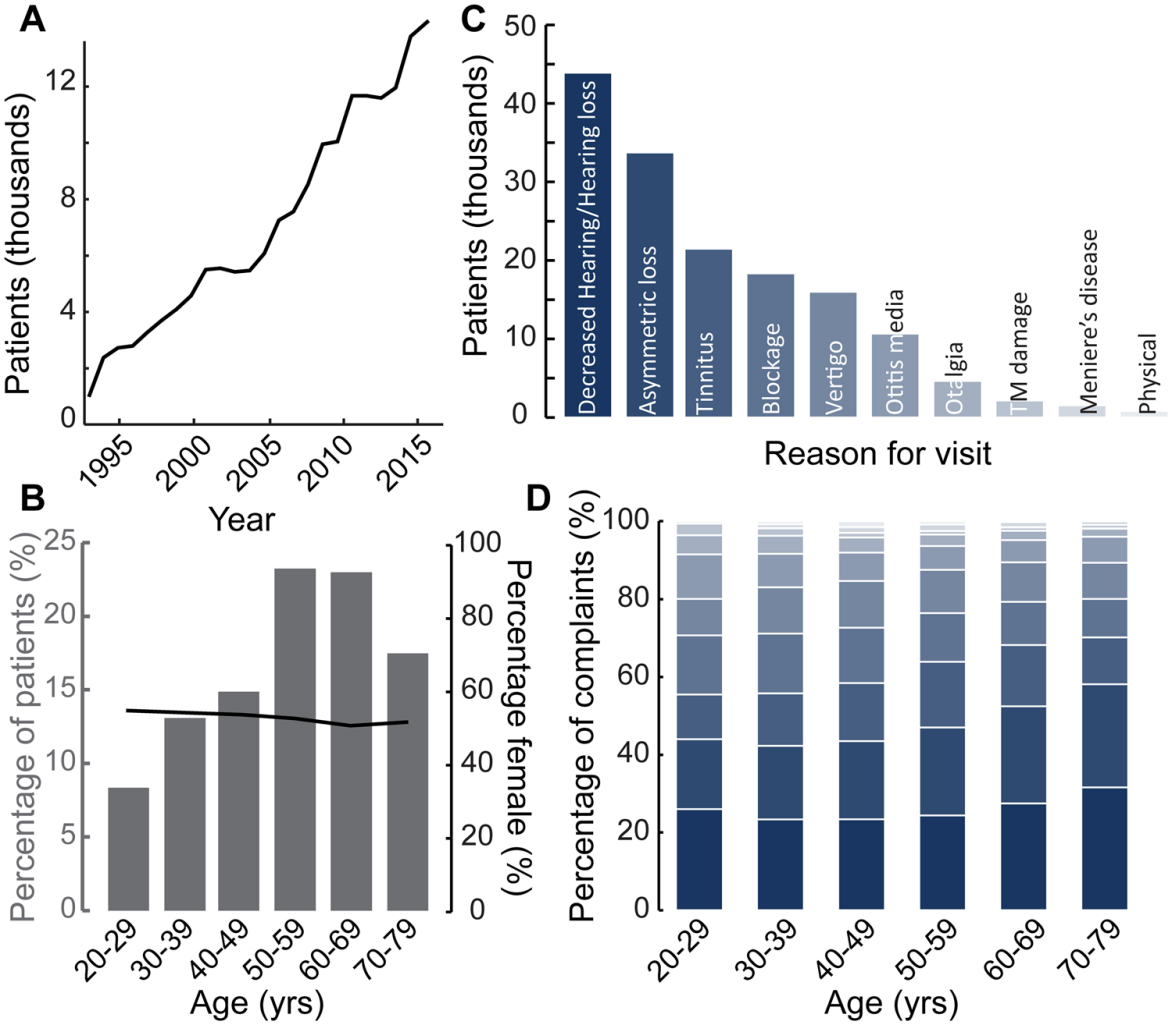
Patient demographics of the Massachusetts Eye and Ear audiological database. (**A**) Number of eligible patients (in thousands) included in the study, as a function of test year. (**B**) Distribution of number of patients (gray axis) and self-reported patient sex (black axis) as a function of age range. (**C**) Number of patients (in thousands) visiting the clinic for each of the top ten reasons for visit. (**D**) Proportional distribution of the top ten complaints, as a function of age range. Color scheme similar to (**C**). TM = Tympanic Membrane.

We compiled pure tone audiometry data collected from 116,400 patients from the MEE Audiology database between 1993 and 2016. The availability of patient records grew over time, hitting a high mark in 2016 with 14,351 patients (Fig. 2A). There was a steady increase in the percentage of patients in the study as a function of age up to 69 years, with patients over 50 comprising 63% of the total population (Fig. 2B, gray bars). The sex distribution remained largely balanced and stable as a function of age, ranging between 51–54% female (Fig. 2B, black line). Between 2000 and 2016, we identified 106,787 cases where the clinical notes indicated a primary reason for visit. Of these patients, the most common reasons for visit as recorded by the audiologist were “decreased hearing” or “hearing loss”, followed by “asymmetric hearing loss”. The other most common reasons for visit in decreasing order were “tinnitus”, “aural fullness” or “blockage”, “vertigo” or “dizziness”, “otitis media”, “pain” or “otalgia”, “perforated” or “damaged tympanic membrane”, “Meniere’s disease” or routine “physical” (Fig. 2C). It is to be noted that the reason for visit was not necessarily exclusive except between “hearing loss/decreased hearing” and “asymmetric hearing loss”, so some patients could appear in multiple other categories. Looking at proportional changes in the ten most common reasons for visit as a function of age (Fig. 2D), there was an uptick in “decreased hearing” or “hearing loss” from 26% in the 20–29 age group to 32% in septuagenarians. “Asymmetric hearing loss” also increased from 18% to 27% in the same age ranges. “Tinnitus” occurrence rates ranged from 11–17%, peaking for patients between 50 and 59 years. “Vertigo” peaked at 12% for patients aged 40–49 and “Meniere’s disease” at 2% for patients between 50 and 59 years. All other reasons showed a steady decline with age (Fig. 2D).

### Classification of patient audiograms based on existing phenotypes

**Figure 3:**
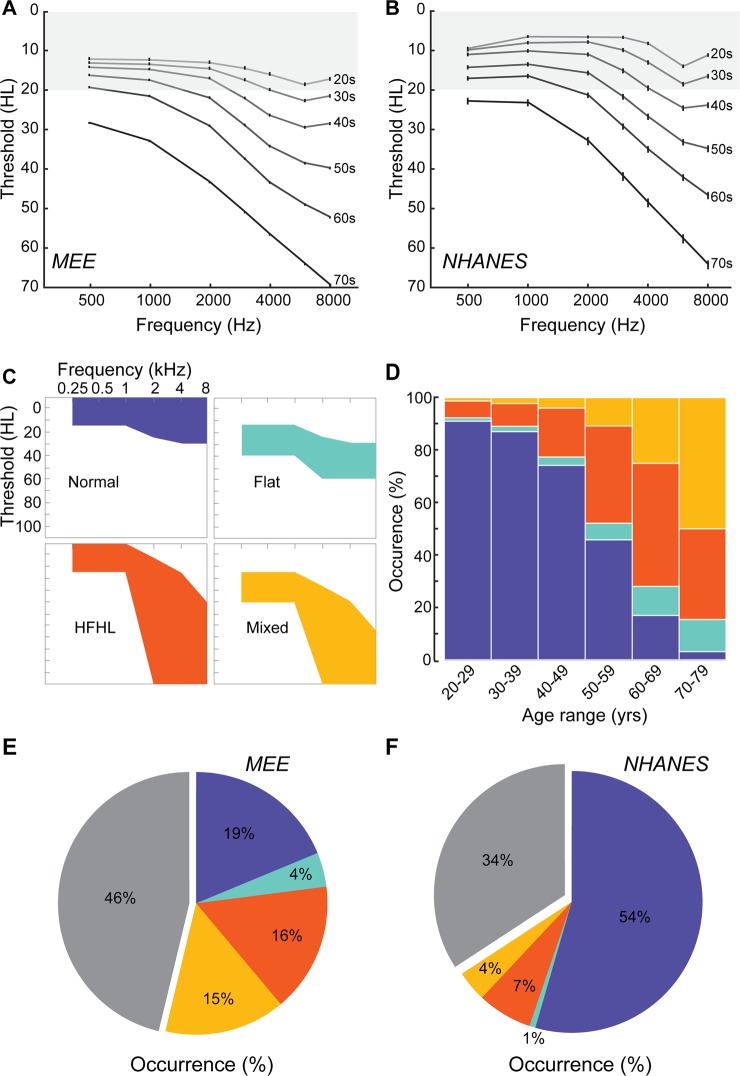
Classification of audiograms based on categorical phenotypes. (**A**) Average audiograms per decade of life in patients from the MEE database. (**B**) Average audiograms per decade of life in subjects from the normative NHANES database. (**C**) Threshold criteria used to classify each of the four audiometric phenotypes – Normal (blue), Flat loss (teal), High frequency hearing loss (HFHL, orange) and Mixed sensorineural loss (yellow). (**D**) Distribution of each phenotype within the classified population, as a function of age in the MEE dataset. (**E,F**) Percentage of each phenotype, Normal, Flat loss, HFHL and Mixed loss as a proportion of all eligible patients, as well as unclassified (gray) audiograms from the MEE dataset (**E**) and NHANES dataset (**F**). Error bars represent SEM.

After first excluding cases with conductive hearing loss, we plotted the average audiogram per decade of life among patients at MEE. Figure 3A shows a gradual decline in hearing thresholds above 2 kHz with age, combined with a loss of thresholds at lower frequencies in patients in their sixth and seventh decade of life. We compared these average audiograms to those obtained from a publicly available normative dataset from the NHANES survey (Fig. 3B). With the exception of some sparing of low-frequency thresholds in the NHANES cohort, audiograms changed similarly over age whether measured from community participants or patients at a large hearing health clinic, which underscores the need for more sensitive measures of audiogram changes across the lifespan.

To more closely examine the prevalence of hearing loss patterns over age, we grouped audiograms into the normal, flat, HFHL and mixed sensorineural hearing loss profiles using the criteria established in prior publications^9,10^. We decreased the threshold limits for frequencies <2 kHz for the normal and HFHL types compared to previous studies to be slightly less restrictive in our classification criteria (Fig. 3C, Table 1). Audiograms were required to conform to the threshold ranges for all frequency regions (i.e., be completely within the shaded region) to be classified into each phenotype.

**Table 1:** Threshold criteria listed by pure tone frequency for classification of audiograms into four general phenotypes.

	<1 KHz		2 kHz		4 kHz		8 kHz	
	Upper	Lower	Upper	Lower	Upper	Lower	Upper	Lower
Normal	−10	15	−10	25	−10	30	−10	30
Flat	20	40	25	60	30	60	30	60
HFHL	−10	15	5	85	15	120	40	120
Mixed	15	40	25	85	40	120	65	120

In the MEE database, the likelihood of patients presenting with normal audiograms declined precipitously between 20–29 (>90%) and the oldest age category (70–79, <3%; Fig. 3D). Aging was associated with an increase in the percentage of HFHL and mixed phenotypes. HFHL phenotype peaked at 46% of the patient population in the 60–69 years age range, while the mixed phenotype and flat hearing loss phenotypes monotonically increased in prevalence with age (Fig. 3D). These trends were consistent with previous published literature regarding the characteristics of these audiogram phenotypes^10^. Looking at the proportion of these audiogram phenotypes within the eligible patient population of this study, normal audiograms comprised 19% of the population, the flat loss phenotype comprised 4%, the high frequency hearing loss comprised 16% and the mixed phenotype comprised 15% (Fig. 3E). Overall, 54% of the audiograms from the database could be classified within one of these four types, with 46% of the audiograms remaining unclassified.

Grouping audiogram records from the normative NHANES database into these four canonical types, accounted for 66% of the underlying data (Fig. 3F). Further, differences in audiogram configurations between normative and clinical records began to emerge, as the NHANES database contained a higher percentage of normal audiograms (54%), the flat type was only 1% of the population, and the HFHL and mixed types were 7% and 4% of the population respectively. Still, over 34% of the audiograms could not be classified into any of these four categories. These results suggest that classifying audiograms based on these four shapes provide an inadequate basis for capturing inherent audiogram variability, especially from a large and diverse clinical population.

### Establishing audiometric phenotypes using clustering by Gaussian mixture models

Gaussian mixture models (GMM) have proven useful for segmenting other types of clinical data from fMRI or gene expression studies^18,19^. GMM classification assumes that the data comes from a mixture of Gaussian distributions. GMM fits the data to a set of Gaussians, allowing them to present different variances between clusters and dimensions. The GMM then assigns samples to a cluster with a certain probability, the posterior probability, which accounts for variance and the means within and between each cluster to estimate the likelihood that the given sample belongs to a cluster.

**Figure 4:**
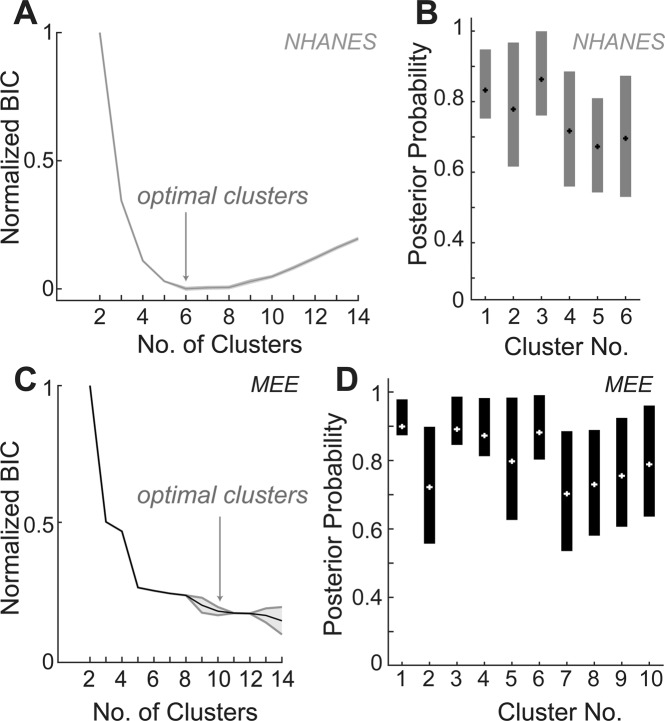
Clustering of audiometric phenotypes based on GMM. (**A**) Bayes Information criteria (BIC) values for each iteration of number of clusters for the NHANES dataset, to determine optimal number of clusters indicated by the gray arrow. (**B**) Posterior probability values of all 6 optimal clusters. (**C**) BIC values for each iteration of number of clusters for the MEE dataset, with optimal number of clusters shown with the gray arrow. (**D**) Posterior probability values for each of the ten optimal clusters identified in C.

We first applied GMM-based segmentation to the NHANES dataset in order to test the validity of our clustering approach on a normative population. In order to converge on the optimal number of clusters, the cluster number was iteratively varied, and the Bayesian information criteria (BIC) value was plotted for each cluster number (Fig. 4A). The best fit of the GMM model was identified by an inflection point in the reduction of the BIC value as more clusters were added. This strategy suggested that six clusters optimally characterized the diversity of audiogram shapes from normative subjects in the NHANES database. The median and the inter-quartile range of the posterior probability values, a measure of the fit of audiogram to its cluster, showed that these phenotypes all had reasonable goodness of fit (Fig. 4B). We then applied the same technique to the MEE dataset. Compared to the NHANES dataset, the MEE dataset showed greater variability in terms of clusters, with the BIC value reaching a minima at 10 clusters (Fig. 4C). The median posterior probability values and inter-quartile ranges for these ten clusters are shown in Fig. 4D.

**Figure 5:**
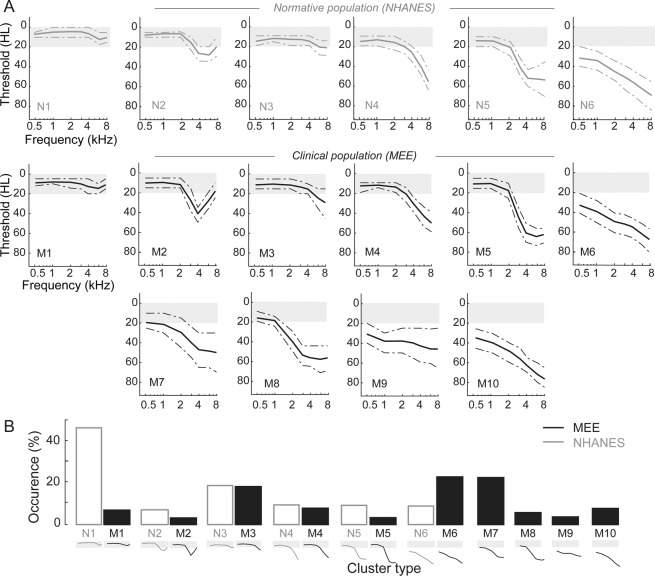
Cluster types and prevalence for NHANES and MEE datasets based on GMM clustering. (**A**) Audiometric phenotypes identified by GMM clustering of the NHANES dataset (top row), and of the MEE dataset that are qualitatively similar (middle row) and additional phenotypes in the MEE dataset (third row). Shaded gray area indicates thresholds better than 20 dB NHL. Solid lines indicate the means and dotted lines indicate the interquartile (50%) range for each phenotype distribution. (**B**) Occurrence of each phenotype in the NHANES (open bars) and the MEE (filled bars) datasets.

The six clusters obtained by the GMM for the NHANES dataset are shown in the first row of Fig. 5A. A qualitative assessment of these shapes revealed a “normal” audiogram (N1), a notch type audiogram (N2) typical of noise damage^13,14^, along with three variants of sloping high frequency hearing loss with inflection points between 2000 and 8000 Hz (N3-N5) and a mixed type with additional losses in the low frequency regions (N6). The GMM from the MEE database identified six audiogram types that were qualitatively similar to the six clusters from the NHANES dataset (Fig. 5A, second row, M1-M6). In addition, there were four more clusters comprising of two additional types of mixed losses with varying degrees of lower frequency hearing loss, a flat loss across all frequencies, and a more extreme high frequency loss beginning at 1000 Hz (Fig. 5A, third row, M7-M10). These audiometric phenotypes reveal additional sources of heterogeneity in our clinical database that are not observed in a cross section of the typical population. Comparing the prevalence of these types between the two datasets, the NHANES dataset contained a much greater proportion of normal audiograms, compared to the MEE database of audiology patients (Fig. 5B). The MEE database however, contained a larger proportion of subjects with hearing loss in frequencies below 2000 Hz.

### Properties of audiometric phenotypes in the MEE audiology database

**Figure 6:**
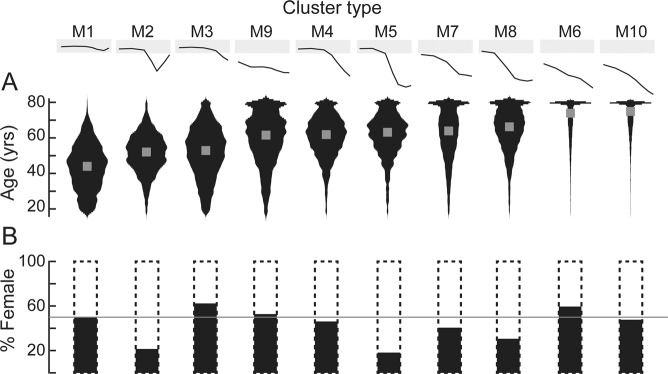
Properties of audiometric phenotypes in the MEE database. (**A**) Violin plots showing the normalized probability distribution of ages for each audiogram type (black) as well as the median age for each type (gray square). (**B**) Percentage of females within each audiogram type. Gray horizontal line indicates 50%. M1-M10 indicate audiogram types shown in Fig. 5.

To relate audiogram cluster to patient age, data were visualized with normalized probability density plots (i.e., violin plots) that present the median value (Fig. 6A, gray square) as well as the variance. The normal (M1) and notched (M2) audiogram types, had the lowest median ages of 44 and 52 years, respectively. The median age of the other clusters increased primarily as a function of the degree of hearing loss, first in the high frequency region and subsequently in the low frequency region. The only exception was M9, where hearing loss was present across all frequencies, which had a median age of 62 years. The two clusters M6 and M10 with the oldest median ages of 74 and 75 years had threshold losses in both the low frequency regions and sloping loss of varying degrees in the high frequency region. Their distribution plots also suggest that the patients in these groups were primarily between 70–80 years old.

Most audiogram shapes showed approximately balanced (±15%) representation between males and females (Fig. 6B), with the exception of three audiogram profiles classically associated with high levels of environmental noise exposure: the 4000 Hz notched audiogram (M2, 21% female) and two variations on the steeply sloping HFHL audiograms (M5 and M8, 18% and 30% female, respectively).

**Figure 7:**
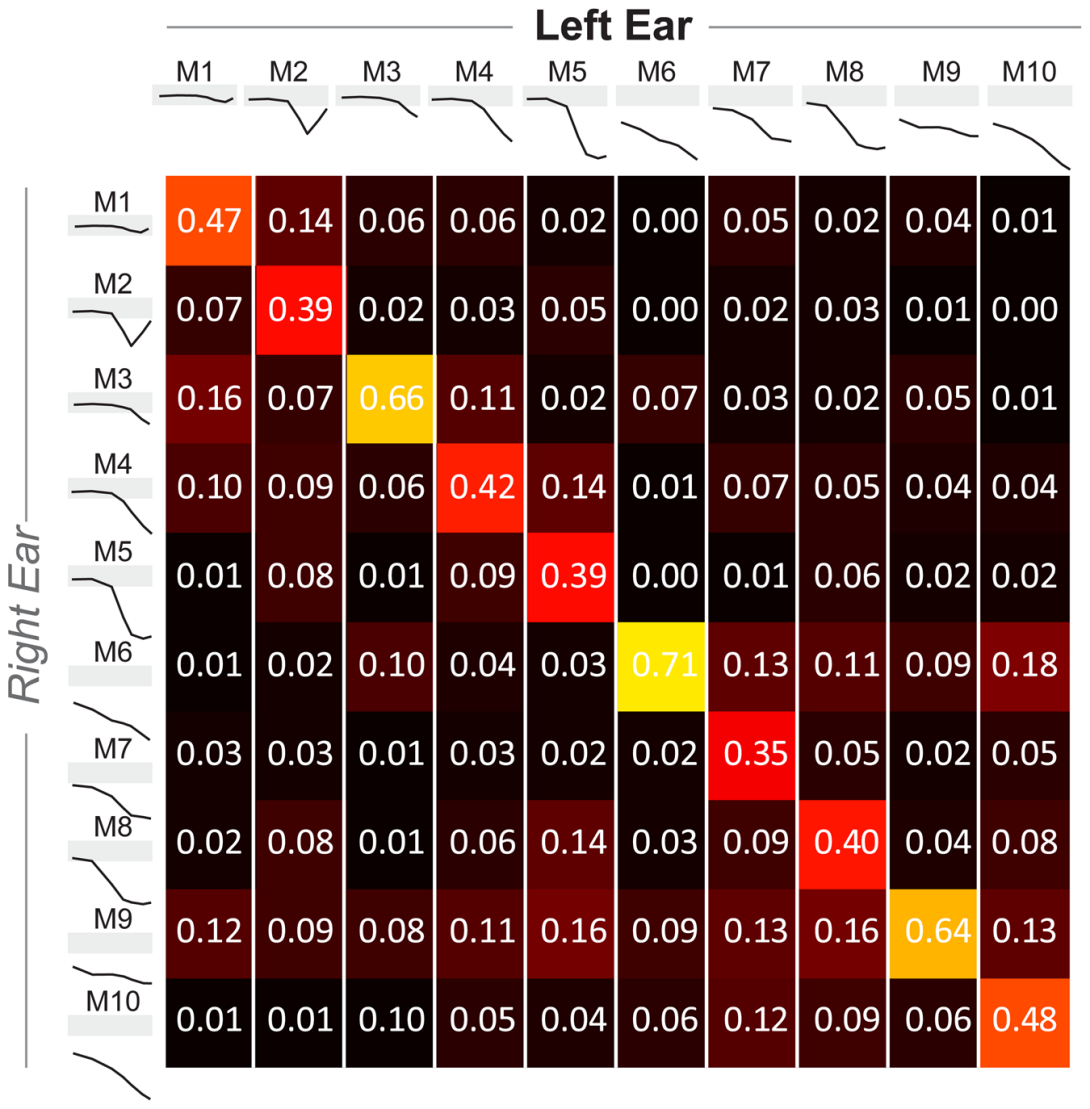
Co-occurrence of audiogram types between left and right ears. Probability of occurrence for the audiogram type in the right ear, given the audiogram type in the left ear. Warmer colors indicate greater percentage of occurrence, with the numerical probabilities shown in white within each box. M1-M10 indicate audiogram types shown in Fig. 5.

These clustering analyses were done using both left and right audiograms, where each audiogram was treated as an independent entity. In order to investigate the between-ear symmetry of these types of audiograms, we subsequently analyzed left and right audiograms separately, and plotted the conditional probability of the right ear occurring in one cluster-type of a patient, given the cluster-type of the left ear. Figure 7 is to be read vertically, with each column showing the conditional probability of the right ear cluster, given the left ear cluster. In most cases, the cluster assignments were between the left and right ears. Clusters with predominantly normal thresholds (M1, M3), flat threshold loss (M9) or mixed types (M6, M10) had the most symmetry. Cluster types with a notch-type audiogram (M2) or steeply sloping high frequency loss (M4, M5, M7, M8) had the least co-occurrence between ears, likely reflecting asymmetric noise damage. When cluster assignments differed, assignments were usually made to a cluster identifying a similarly shaped audiogram, such as between M6, M7 and M10.

## Discussion

Audiometric profiles have traditionally been phenotyped based on presumed underlying histopathology, either from animal studies or human temporal bones^6–12,20–22^. The general shapes that are prevalent fall broadly into categories of normal, sloping high frequency loss and a flat loss across all frequencies. However, a large number of audiogram types either do not fit into these clearly defined types, remain unclassified, or are presumed to be of mixed origin^6,10^. One way to counter the degree of uncertainty of classification is to look at exemplars of each type using human observers and build a classifier to place other audiograms into these types^10^. Alternatively, audiograms may best conform to a heterogeneous continuum rather than discretized clusters, which has raised the question of whether the notion of an audiogram “type” should itself be re-examined^16^. In this study, we addressed this question using over 130,000 audiograms of English speaking patients between 18 and 80 years of age, from the Massachusetts Eye and Ear audiology database over a 24-year period.

In the audiology database at MEE, patients over the age of 50 constituted 63% of the total patient population (Fig. 2B). The gender distribution was slightly leaning towards greater females (Fig. 2B), likely reflecting the gender distribution of the New England region served by our clinic (www.census.gov). The primary reasons that patients visited the clinic included symmetric and asymmetric hearing loss, tinnitus, aural blockage and otitis media (Fig. 2C), with various forms of hearing loss reported in more than 70,000 patients, and tinnitus reported in over 20,000 patients over a 16-year period from 2000 onwards. Hence some form of hearing loss constituted 51% of the visits to the clinic. Tinnitus was the next most prevalent reason after hearing loss and constituted 14% of the visits in the overall patient population, consistent with previous reports about the prevalence of tinnitus^23–25^.

Within the patient population at MEE, the average audiogram per decade of life showed a gradual decrease in hearing thresholds, initially in frequencies above 2 kHz, followed by lower frequencies in the oldest age groups (Fig. 3A). These distributions were largely similar to normative audiograms from the publicly available NHANES database (Fig. 3B), suggesting that mean audiograms are not sensitive to differences between the two populations. Under categorical classification, within the MEE population, the proportion of normal audiograms decreased as a function of age, while the prevalence of hearing loss increased (Fig. 3D). In particular, while the flat type stayed reasonably constant at age groups >60 years, the HFHL and the mixed type showed a steady increase in proportion as a function of age. Normal audiograms made up 19% of all audiograms in our dataset, larger than what has been previously reported^10^. This is likely due, in part, to the difference in age-ranges considered. Previous studies have assessed changes in audiogram shapes primarily in aged listeners over 50 years old, while we included patients from 18–80 years of age.

Overall, only 54% of the audiograms fell into these distinct categories based on previously published criteria (Fig. 3E). Comparing these classifications to audiograms from the NHANES database, the categorical classification system fared better, but still excluded a third of the audiograms (Fig. 3F). This illustrated the need for a data driven clustering method that was agnostic to presumed underlying otopathology, to better capture the diversity in audiograms, especially in our clinical database.

Previous studies using data driven methods have approached this problem of clustering with the use of hierarchical clustering^16^. Hierarchical clustering has some limitations when used to cluster audiograms. The shortest mutual distance used as the linkage criterion does not account for the ‘stereotype’ (centroid) of each cluster when grouping samples or the overall distribution of samples within each cluster, and it is therefore prone to creating ‘chains’ of samples as groups. In the application to audiograms, this may lead to linking samples that present progressive changes between each other into one same category. In addition, hierarchical clustering suffers from missing the global organization of the data as it optimizes locally in each grouping step, an issue that is enhanced if the sample size is too large. Other techniques such as k-means clustering perform a global optimization to segregate samples into partitions or clusters, but then assigns samples to a cluster considering only how different it is to the mean of that cluster. It further assumes that the groups present the same variance across all the dimensions of the data and share the same covariance matrix. It therefore presents the limitation of generating equally sized groups that look ‘spherical’ in their variance. This would imply that all the threshold measurements in each frequency within each cluster should present the same variability, and that all the clusters share this same variance. In order to account for these limitations, we used a Gaussian mixture models (GMM)-based approach to cluster the audiograms in this study. As opposed to hierarchical or k-means clustering, GMM attempts to find a global optimum, considering how the entire dataset fits the model in each optimization step, and further takes into account the entire distribution of samples and unequal variances of each cluster. The choice for the optimal number of clusters for each dataset using the GMM was determined using the BIC values, a more conservative criterion than the AIC which implicitly penalizes model complexity and thus minimizes overfitting of the data.

We extended our clustering approach by applying the GMM to the normative dataset from the NHANES database. Clustering this database resulted in more homogeneity in audiogram profiles, with the model converging on six optimal clusters. The shapes of these clusters were largely in agreement with previous studies using normative populations^10,12,16^. When then applied to the audiograms in the MEE database, the GMM identified several audiogram variants that were not found in the NHANES database, as expected from the preponderance of older subjects with significant hearing loss in this clinical population (Fig. 5). Broadly speaking these clusters included patients with “normal” thresholds (M1), “notch” type audiograms (M2) and audiograms with varying degrees of high frequency hearing loss with deflection points at 8 kHz (M3), 4 kHz (M4), 2 kHz (M5) and 1 kHz (M8). In addition, there was a flat-type audiogram with loss across all frequencies (M9), and audiogram types with varying degrees of low frequency loss in addition to the sloping high frequency hearing loss (M6, M7, M10). Patients with these mixed types tended to be, on average, older in age, while normal and notch types tended to be the younger. Some cluster types like M6 and M10 looked qualitatively similar to one another, except for a slightly greater loss in the higher frequencies. However, the GMM algorithm placed them as separate clusters, and some differences were also found between these clusters in their gender distribution, with a greater percentage of males in the type with greater high frequency loss (Fig. 6). Other audiogram shapes have been traditionally associated with noise overexposure and sensorineural hearing loss, specifically the 4000 Hz notch type and the steeply sloping high frequency hearing loss type^12,15,26^. In this study, these shapes also tended to be predominantly male, likely reflecting historical differences in occupational noise exposure between men and women^27,28^.

One limitation of using data-driven segmentation methods is that it is harder to assign underlying causes to each audiogram cluster. The GMM identified several phenotypes that have been previously identified with specific otopathologies. Degrees of sloping high frequency hearing loss are thought to be sensorineural in origin, caused due to a combination of aging and noise exposure^11^. The flat loss across all frequencies is thought to be due to metabolic changes in the stria vascularis^7,8^. Other phenotypes such as the cochlear conductive type have been proposed when thresholds do not correlate with the underlying histopathology^6^. However, it is to be noted that most of these changes were theorized using light microscopy techniques which may miss critical elements in the cochlea. Recent studies have cast doubt on the underlying causes for these hearing profiles, and suggest that inner hair cell loss, outer hair cell loss, strial losses and spiral ganglion loss all work in concert to produce the different audiometric phenotypes like sloping hearing loss or flat loss^17,29,30^. Further complications like changes occurring in hearing during the time between the last audiogram and the post-mortem collection and analysis, as well as the loss of cochlear synapses which render those auditory nerve fibers useless even when the spiral ganglion cells themselves remain, all complicate our understanding of otopathology. We chose to apply these data-driven methods in order to be agnostic to these changes. The unbiased, data-driven GMM independently converged on many of the same cluster types as these *a priori* approaches, confirming that they may reflect discrete underlying etiologies. However, the data-driven clustering identified substantial heterogeneity within types, underscoring the need to pull in additional markers of hearing loss such as environmental overexposure history, or genetic risk factors to arrive at a more comprehensive classification of hearing status^31–34^. We performed a linear interpolation of threshold data at 3 and 6 kHz in the MEE database, in order to provide the clustering algorithms with a balanced dataset comparable to NHANES. Measuring thresholds at inter-octave frequencies is not yet standard clinical practice, but can be useful for understanding hearing complaints like tinnitus, which often accompanies steep changes in pure tone thresholds in narrow frequency ranges^35^. Inclusion of such frequencies in standard audiometric testing may provide additional information regarding heterogeneity of audiometric phenotypes in future studies.

Both M1 and to some extent, M3 would typically be considered normal audiograms. The amount of co-occurrence between the left and right ears for these two types (Fig. 7) suggests that a large percentage of people with bilaterally normal audiograms are coming to the clinic complaining of hearing problems. This adds to the growing literature showing that the audiogram in itself is not sufficient to capture hearing difficulties experienced by people and demonstrates the need for more sensitive tests in the clinic that capture the real-world communication difficulties experienced by patients^36–38^.
